# Evaluation of Commercial RNA Extraction Protocols for Avian Influenza Virus Using Nanopore Metagenomic Sequencing

**DOI:** 10.3390/v16091429

**Published:** 2024-09-07

**Authors:** Maria Chaves, Amro Hashish, Onyekachukwu Osemeke, Yuko Sato, David L. Suarez, Mohamed El-Gazzar

**Affiliations:** 1 Department of Veterinary Diagnostic and Production Animal Medicine, College of Veterinary Medicine, Iowa State University, Ames, IA 50011, USA; mpeixoto@iastate.edu (M.C.); hashish@iastate.edu (A.H.); oosemeke@iastate.edu (O.O.); ysato@iastate.edu (Y.S.); 2 National Laboratory for Veterinary Quality Control on Poultry Production, Giza 12618, Egypt; 3 US National Poultry Research Center, Agricultural Research Service, US Department of Agriculture, Athens, GA 30605, USA; david.suarez@usda.gov

**Keywords:** avian influenza, metagenomic sequencing, nanopore sequencing, nucleic acid extraction

## Abstract

Avian influenza virus (AIV) is a significant threat to the poultry industry, necessitating rapid and accurate diagnosis. The current AIV diagnostic process relies on virus identification via real-time reverse transcription–polymerase chain reaction (rRT-PCR). Subsequently, the virus is further characterized using genome sequencing. This two-step diagnostic process takes days to weeks, but it can be expedited by using novel sequencing technologies. We aim to optimize and validate nucleic acid extraction as the first step to establishing Oxford Nanopore Technologies (ONT) as a rapid diagnostic tool for identifying and characterizing AIV from clinical samples. This study compared four commercially available RNA extraction protocols using AIV-known-positive clinical samples. The extracted RNA was evaluated using total RNA concentration, viral copies as measured by rRT-PCR, and purity as measured by a 260/280 absorbance ratio. After NGS testing, the number of total and influenza-specific reads and quality scores of the generated sequences were assessed. The results showed that no protocol outperformed the others on all parameters measured; however, the magnetic particle-based method was the most consistent regarding C_T_ value, purity, total yield, and AIV reads, and it was less error-prone. This study highlights how different RNA extraction protocols influence ONT sequencing performance.

## 1. Introduction

Avian influenza is an infectious disease that significantly impacts human and animal health and the economy worldwide [1]. The disease is caused by a segmented negative-sense RNA virus, a member of the influenza A virus (IAV) genus (Orthomyxoviridae family), and it is classified into different subtypes based on its surface glycoproteins—hemagglutinin (HA) and neuraminidase (NA) [2]. Wild waterfowl are the main reservoir for viral strains and are a source of introduction of avian influenza virus (AIV) outbreaks to poultry. The 2015–2016 highly pathogenic avian influenza (HPAI) outbreak was considered the most impactful foreign animal disease in U.S. history at the time. However, the current 2022–2024 outbreak has already surpassed this outbreak in the number of affected animals, including dairy cattle [3,4].

The antigenic drift and shift of avian influenza viruses (AIVs) are a significant concern to both animal and human health, leading to constant viral monitoring at a global scale [1,5,6,7,8]. Current monitoring strategies focus on tracking viral mutations by performing molecular analyses, such as rRT-PCR (quantitative real-time reverse-transcription polymerase chain reaction) and the amplicon-based gene sequencing of its surface glycoproteins HA and NA [9]. Additionally, there is a rising demand and expectation for full-length viral genome characterization to comprehend the viral evolution and epidemiology of newly emerging and re-emerging AIV strains. Phylogenetic analysis of all genomic segments of the Eurasian A/goose/Guangdong/1/1996 (GsGd) lineage detected in late 2021 in North America demonstrated the transatlantic spread of the H5N1 strain from Europe, associated with wild bird migration [10,11].

Improved Sanger sequencing and the introduction of second-generation sequencing platforms allow the comprehensive genomic characterization of AIVs from isolates and clinical samples, contributing to a better understanding of human and animal infectious diseases [12,13,14,15,16]. However, these current technologies have limitations, such as a long turn-around time, extensive protocols, high cost per sample, and the need for large bench equipment [17,18]. On the other hand, the advent of the Oxford Nanopore Technology (ONT) platform, a third-generation sequencing platform, circumvents these limitations and further facilitates the use of whole genome sequencing (WGS) in infectious disease diagnostics [19]. ONT has provided cost- and time-effective solutions for sequencing workflows that require a lower cost per sample, fewer initial investments, and less complex library preparation procedures, leading to a shorter time from sample to results. Additionally, the sequencing can be run on a USB-connected sequencer using laptop computers and analyzed with user-friendly data analysis tools [20,21,22]. ONT’s portable devices, such as the MinION, have enabled the remote, real-time analysis of sequencing data outside the laboratory setting [23,24]. However, the technology has several limitations, such as a higher error rate compared to short-read sequencing [25], constant updates of kits and software, and the short shelf life of flow cells and reagents, making selecting the most appropriate workflow more challenging [26].

The features of ONT, particularly the real-time access to the sequence results, make the technology suitable for metagenomic next-generation sequencing (mgNGS), in which AIV nucleic acid can quickly be identified and characterized directly from clinical samples. The success of viral WGS using ONT directly from clinical samples primarily hinges on a multi-step preparation process. This process includes nucleic acid extraction, sequencing library preparation, and the subsequent assembly and analysis of the generated reads, culminating in sequence results [27]. Nucleic acid extraction is the first and critical step to maximize the applicability of ONT in diagnostics settings. There are multiple available nucleic acid extraction protocols, and the selection of the optimal method is complex and depends on the sample type and the desired outcome [28]. Therefore, multiple studies have been conducted to assess the impact of different extraction procedures in nucleic acid recovery from clinical samples using ONT [27,29,30,31]. Previous work that analyzed complex microbial diversity and the metagenomic use of Nanopore sequencing has shown that the yield and length of DNA extracted by different protocols impacted the microbial abundance from urine and tongue dorsum samples [29,30]. Another study has assessed the impact of different extraction procedures in AIV RNA recovery from nasal samples using the Illumina MiSeq platform. The research compared five extraction protocols, and the protocol MagNA pure compact RNA isolation (automated extraction based on the magnetic isolation of nucleic acids) consistently gave the best results in AIV segment coverage depth at different time points after inoculating the virus in ferrets [32].

Even though similar work has been conducted on comparing different extraction protocols for AIV recovery from clinical samples using Illumina (short-read sequencing), no data are available for a similar evaluation using ONT (long-read sequencing). Therefore, the objective of this study is to compare four commercially available and commonly used RNA extraction protocols (magnetic particle-based, spin column, liquid-phase separation, and the enzymatic method) and evaluate their performance in terms of concentration and purity, followed by metagenomic nanopore sequencing for their comprehensive evaluation.

## 2. Materials and Methods

### 2.1. Clinical Samples

The samples used in the study (*n* = 24) were received at the Iowa State University Veterinary Diagnostic Laboratory (ISU-VDL) in 2022. The samples were confirmed positive at the National Veterinary Services Laboratory (NVSL) in Ames, IA, for Influenza A by (rRT-PCR) targeting Eurasian lineage goose Guangdong H5 clade 2.3.4.4b. As a tier 1 member of the National Animal Health Laboratory Network (NAHLN), the samples received in ISU-VDL were handled and disposed of according to the Guidelines for Avian Influenza viruses established by the U.S. Department of Agriculture and Animal and Plant Health and Inspection Service (USDA-APHIS). A complete list of samples and their details is presented in Table 1. All samples were further flushed and manually homogenized using 3 to 5 mL of phosphate-buffered saline (PBS) to elute the tissues. PBS was selected in our methodology because it is a widely available transport media, it is compatible with ONT downstream applications (no interference in the following steps), non-toxic, presents buffering capability and pH stability, and it has been widely used for AIV transport and storage [33,34,35]. The liquid from each sample was then collected and aliquoted into 1.5 mL Eppendorf tubes and briefly centrifuged at 12,000 rpm for 30 s. Then, total RNA was extracted from the supernatant obtained from the samples. 

Nucleic acid extraction was carried out twice on each sample using each protocol (two sections), resulting in 48 individual observations per kit. These two sets of experiments were conducted to test the repeatability of the results (Supplementary Figure S1).

### 2.2. Nucleic Acid Extraction Kits Used in This Study

**Figure 1:**
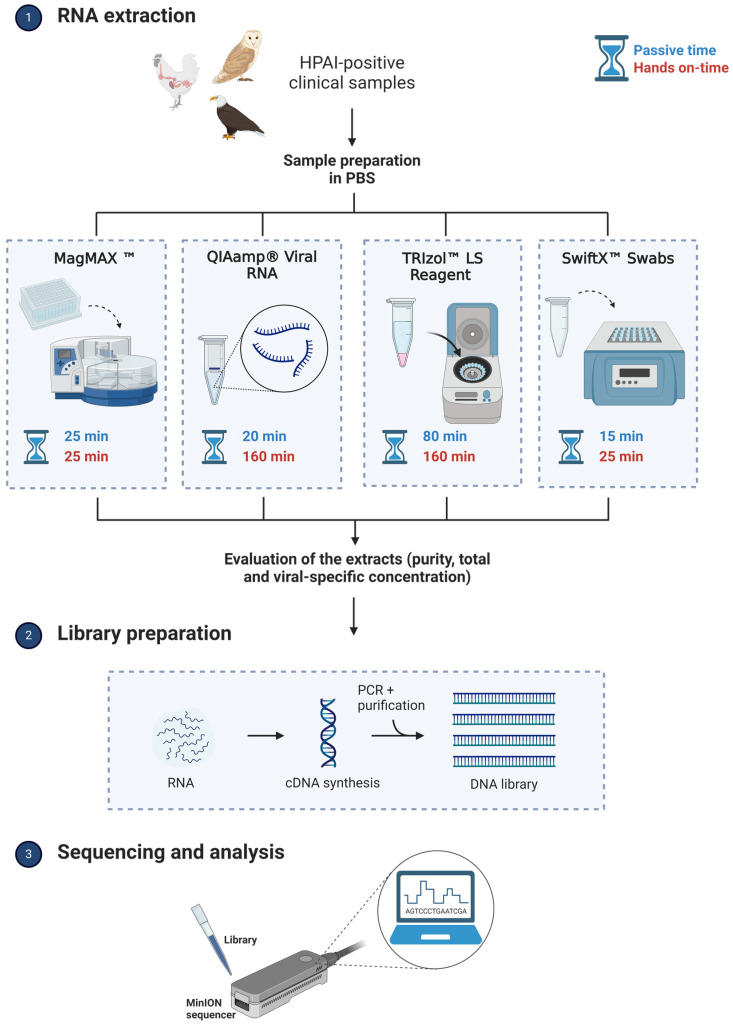
Experimental workflow. The total time (spent extracting 24 samples) was split into passive and hands-on time for each protocol.

Four different nucleic acid extraction methods were tested using different commercial extraction kits: magnetic particle-based, silica column, liquid-phase separation, and enzymatic extraction methods (Table 2). Each method is fully described below, and the experimental workflow is shown in Figure 1.

(1) Method (A): Magnetic particle-based method using MagMAX™ Pathogen RNA/DNA Kit (Thermo Fisher Scientific, Waltham, MA, USA)

One aliquot of the supernatant from each sample (100 μL) was used to extract the viral RNA following the manufacturer’s instructions. Briefly, the steps consisted of the lysis of the samples using 240 μL of the lysis buffer for bead beating, two washing steps, and a final elution step in 90 μL. For the lysis buffer preparation, RNA carrier was not included to avoid sequencing the extraneous RNA. Extraction was performed using the KingFisher™ Flex Purification System (Thermo Fisher Scientific, Waltham, MA, USA).

(2) Method (B): Silica column using QIAamp^®^ Viral RNA (QIAGEN, Hilden, Germany):

A 140 μL aliquot from the supernatant of each sample was used to extract viral RNA following the manufacturer’s instructions with a few modifications. First, the carrier RNA was not added to the viral lysis (AVL) buffer to avoid sequencing the extraneous RNA. Additionally, the elution step was modified to increase the RNA elution volume, and it consisted of (1) the addition of 30 μL of the elution buffer, (2) incubation at room temperature for one minute, and (3) centrifuging at 8000 rpm for one minute. These sequential steps were performed twice, resulting in a 60 μL total eluate.

(3) Method (C): Liquid-phase separation using TRIzol™ LS Reagent (Invitrogen, Waltham, MA, USA)

A 250 μL aliquot of the supernatant from each sample was homogenized with 750 μL of TRIzol reagent for cell lysis, followed by chloroform addition and phase separation (a lower red phenol–chloroform phase, white interphase, and a colorless upper aqueous phase containing the RNA). Following this step, the RNA was collected from the aqueous phase, precipitated in 500 μL of isopropanol, and centrifugation was performed for 15 min at 18,400× *g* at 4 °C for pellet formation. The pellet was resuspended in 1 mL of 75% ethanol and centrifuged for 5 min at 7500× *g* at 4 °C. The dried pellet was eluted in 50 μL of nuclease-free water.

(4) Method (D) Enzymatic extraction SwiftX™ Swabs (Xpedite Diagnostics GmbH, Hallbergmoos, Germany)

The extraction buffer (component E) was activated by dissolving two enzyme blends (components C and P) following the manufacturer’s instructions. Subsequently, extraction was performed from the liquid samples by mixing 100 μL of the activated component E with 100 μL of each sample. The solution was incubated for 15 min at 90 °C and then cooled down and homogenized by vortexing the final elution. 

Extracted RNA from all methods was kept at 4 °C till further rRT-PCR testing and evaluation. All analyses were performed within a week to preserve RNA integrity.

### 2.3. Evaluation of the Extracted RNA

#### 2.3.1. Viral-Specific RNA Quantification

To assess the performance of each method in detecting viral-specific RNA, samples extracted from each method were tested for IAV using rRT-PCR targeting the virus Matrix gene. Testing was performed on the ABI 7500 Fast Real-Time PCR system (Applied Biosystems, Foster City, CA, USA) using a 20 μL total reaction volume containing 8 μL of RNA extract, 0.8 μL of Influenza Virus primers and probe mix (Avian Influenza Virus RNA Test kit—VetMAX^TM^ Gold AIV Detection kit, Thermo Fisher Scientific, Waltham, MA, USA), 5 μL TaqMan Fast Virus 1-Step Master Mix (Life Technologies. Carlsbad, CA, USA), and 6.2 μL of nuclease-free water. The cycling conditions were 50 °C for 5 min and 95 °C for 20 s, followed by 40 cycles of 95 °C for 15 s and 60 °C for 1 min. Samples with C_T_ (cycle threshold) values equal to or higher than 40 were considered negative. The C_T_ value was used to approximate the IAV RNA concentration within each sample. The rRT-PCR detection rate was used as an evaluation parameter. This was a binary classification: samples with a C_T_ value less than 40 were assigned the value 1 (positive), while those with a C_T_ value of 40 or higher were assigned the value 0 (negative).

#### 2.3.2. Total RNA Quantification and Purity Evaluation

The RNA concentration was assessed using the Qubit 4.0 Fluorometer and Qubit™ RNA HS Assay Kit (Thermo Fisher Scientific, Waltham, MA, USA) according to the manufacturer’s protocol. Left-side censoring was established at 0.25 ng/µL for all samples with concentration values below the assay’s limit of detection (0.25 ng/µL). The purity of each extract was verified using the NanoDrop Spectrophotometer (Thermo Fisher Scientific, Waltham, MA, USA) based on an A260/A280 ratio, and sample values between 1.9 and 2.2 were considered to have a high RNA purity.

#### 2.3.3. Generation of MinION Nanopore Libraries, Sequencing, and Data Analysis

After extracting and evaluating the extracts, three samples (sample numbers five, fourteen, and sixteen—Table 1) were selected, extracted by the four protocols (*n* = 12), and used to prepare sequencing libraries. These samples were selected based on their tissue type, with the trachea representing the respiratory tract and the brain representing the neurological system, two systems commonly affected by the highly pathogenic avian influenza. Additionally, the selected samples had a variety of total and viral-specific (rRT-PCR C_T_ value) RNA concentrations (C_T_ values from 11.4 to 23; total RNA concentration from 378 to 1975 ng/µL) to evaluate the performance of ONT across a wide range of concentrations in the tested samples.

Two separate sequencing libraries were prepared with ONT’s PCR-cDNA sequencing–barcoding protocol (SQK-PCB109) following the manufacturer’s instructions. Briefly, the steps included (1) reverse transcription and strand switching, (2) a PCR step and the addition of barcoding to the transcripts, and (3) adapter ligation to the amplified cDNA library. After library preparation, each sequencing library was pooled and loaded onto a separate MinION R9.4.1 (FLO-MIN106D) flow cell (ONT) after priming with the Flow Cell Priming Kit (EXP FLP002) according to the manufacturer’s instructions. The library containing sample 14 was run in a flow cell with 1141 pores, with 100 fmol of the library loaded. In contrast, for samples 5 and 16, the flow cell had 1314 pores, and 260 fmol of the library was loaded. The flow cells ran for 14 and 12 h, respectively, using the MinION Mk1B sequencer. MinKNOW software (21.06.0 version) was used to start and monitor the progress of the sequencing run. Guppy (5.0.11 version) within MinKNOW was used for base calling (fast accuracy base calling mode) and demultiplexing. The quality filtering employed a minimum score of seven, whereas length filtering applied to sequences within the range of 50 to 3000 bases. The generated FASTQ files were further analyzed using the WIMP tool within EPI2ME (Agent version 3.6.2) to calculate the total yield (in millions of bases), read length, read quality, and the number of specific AIV reads generated during the runs, to compare the effect of the four extraction methods on the generated sequences. Additionally, the Nanoplot [36] tool provided by Galaxy software v 22.05 (https://usegalaxy.org/) was used to assess the N50 from the generated FASTQ files. This sequencing approach is a mgNGS without prior enrichment. Although mgNGS is known for its poor sensitivity in viral detection, we chose not to apply any enrichment to ensure a fair comparison between methods.

#### 2.3.4. Protocol Applicability and Sample Type Comparison

Establishing an applicable extraction process with the MinION nanopore sequencing platform will create an easy operational protocol for identifying and characterizing AIV from clinical samples. In our study, we assessed the protocol’s applicability based on several key factors, including its flexibility (such as the ability to extract from various sample types), reproducibility, susceptibility to personal bias, the balance between hands-on and passive time, cost considerations, and input/output volumes. Our study’s primary objective was to identify the most suitable protocol; therefore, we examined the applicability characteristics of the methods to guide our selection. The sample types are another crucial element influencing the extracted RNA and generated reads. As a result, we also compared the performance of the extraction protocols with different tissue types by contrasting the total RNA concentration and rRT-PCR detection rates. 

### 2.4. Statistical Analysis

All statistical analyses were performed on the R statistical software [37]. Tables and figures were used to describe and assess the performance of the extraction protocols; figures were generated using the ggplot2 package [38] in R statistical software. Mixed-effects regression models were used to evaluate statistical differences in the performance of the extraction protocols for each variable assessed.

#### 2.4.1. Statistical Analysis of RNA Quantification and Evaluation of Purity

**Figure 2:**
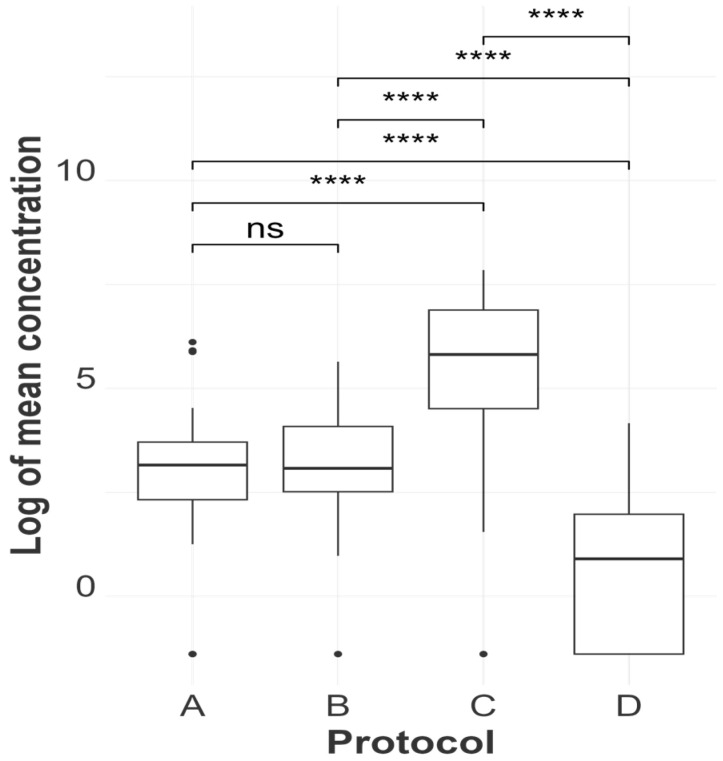
The log mean concentration distribution for each extraction protocol is represented as MagMAX Pathogen RNA/DNA™ (A), QIAamp^®^ Viral RNA (B), TRIzol™ LS Reagent (C), and SwiftX™ Swabs (D). Statistical differences between protocols are indicated atop the boxplots using the results from a mixed-effect regression model (protocol as a fixed effect and tissue type nested within bird as a random effect). No significance “ns”; “****” indicate *p*-values > 0.05 and ≤0.0001, respectively. An alpha value of 0.05 was used, and *p*-values were adjusted using the Sidak method for 6 tests.

**Figure 3:**
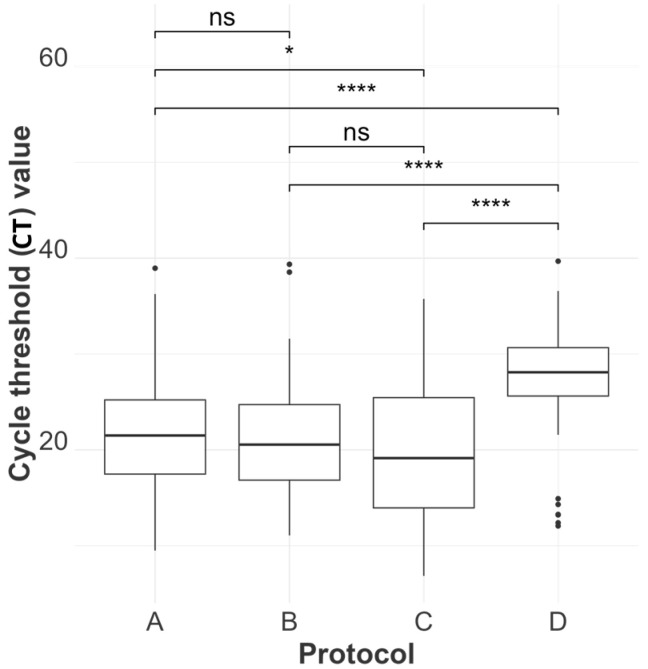
The distribution of cycle threshold values for each extraction protocol is represented as MagMAX Pathogen RNA/DNA™ (A), QIAamp^®^ Viral RNA (B), TRIzol™ LS Reagent (C), and SwiftX™ Swabs (D). Statistical differences between protocols are indicated atop the boxplots using the results from a mixed-effect regression model (C_T_ as the protocol as a fixed effect and tissue type nested within bird as a random effect). No significance “ns”, “*”, and “****” indicate *p*-values > 0.05, ≤0.05, and ≤0.0001, respectively. An alpha value of 0.05 was used, and *p*-values were adjusted using the Sidak method for 6 tests.

**Figure 4:**
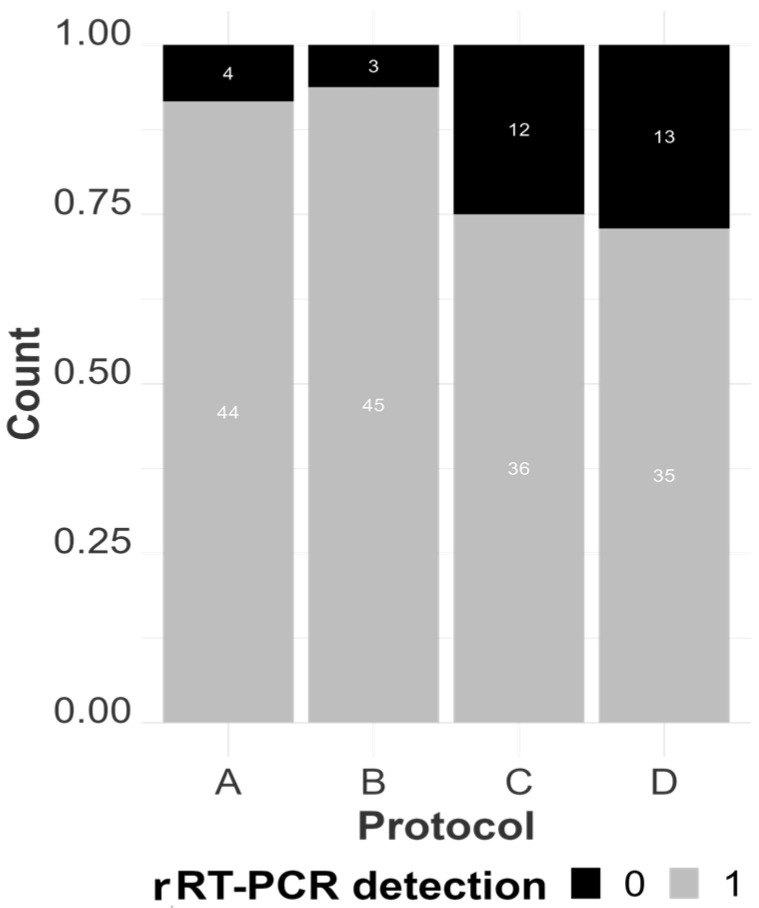
The count of positive samples by rRT-PCR by extraction protocol, represented as MagMAX Pathogen RNA/DNA™ (A), QIAamp^®^ Viral RNA (B), TRIzol™ LS Reagent (C), and SwiftX™ Swabs (D). The total number of evaluated extracts per protocol was 48, considering that we extracted 24 samples in two different sections, resulting in 48 individual results per protocol. An extract has a positive detection (1) when the C_T_ value is less than (<) 40 and a negative detection (0) when the C_T_ value is equal to or higher than 40.

**Figure 5:**
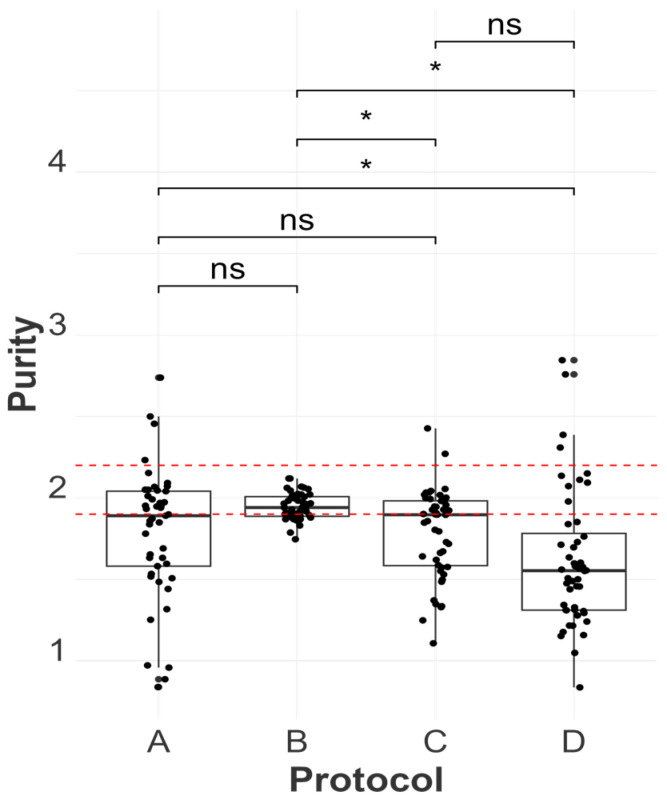
The distribution of purity values for each extraction protocol is represented as MagMAX Pathogen RNA/DNA™ (A), QIAamp^®^ Viral RNA (B), TRIzol™ LS Reagent (C), and SwiftX™ Swabs (D). Statistical differences between protocols are indicated atop the boxplots using the results from a mixed-effect regression model (purity (pure or impure) was the response variable, the extraction protocol was the fixed effect, and tissue type nested within the bird was used as a random effect). “ns” and “*” indicate *p*-values > 0.05 and ≤0.05 respectively. An alpha value of 0.05 was used, and *p*-values were adjusted using the Sidak method for 6 tests. The red dashed lines indicate the range of absorbance ratio values between 1.9 and 2.2 within which an extract is considered pure.

**Figure 6:**
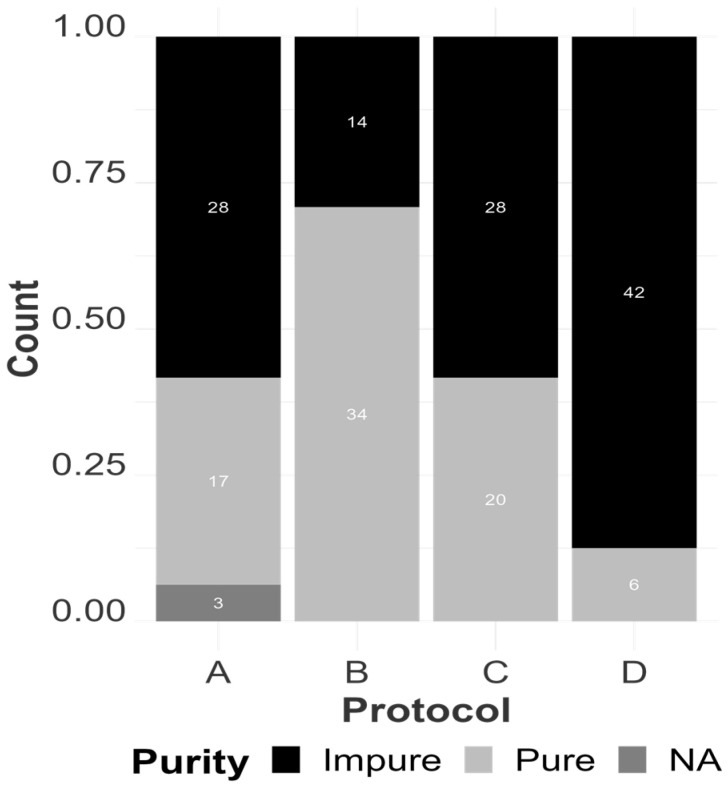
The count (numbers within the bars) of pure and impure extracts by extraction protocol, represented as MagMAX Pathogen RNA/DNA ™ (A), QIAamp^®^ Viral RNA (B), TRIzol™ LS Reagent (C), and SwiftX™ Swabs (D). An extract is considered pure if the absorbance ratio values lie between 1.9 and 2.2. “NA”: purity could not be assessed due to insufficient extract volume.

Table 3, Table S1 and Figure 2, Figure 3, Figure 4, Figure 5 and Figure 6 illustrate and compare the extraction protocols’ performances in quantifying the total and IAV-specific RNA, extract purity, and rRT-PCR detection rates.

Linear mixed regression models were used to assess the least squares mean differences in the log mean RNA concentration and least squares mean differences in C_T_ values between protocols. In these models, the listed variables were each used as response variables, the extraction protocol was the fixed effect, and tissue type nested within each animal ID was used as a random effect to account for sample-specific attributes that could influence the performance of the extraction protocols. 

The values obtained from the purity assessment were discretized into two categorical groups (pure or impure) based on having an A260/A280 ratio value within the range of 1.9–2.2 (inclusive) or not. Similarly, rRT-PCR C_T_ values below 40 were considered positive; otherwise, they were negative, hence also discreet. Logistic mixed regression models were thereafter used to assess purity and IAV rRT-PCR detection rates across the extraction protocols.

#### 2.4.2. Statistical Analysis of the Generated Nanopore Sequencing Data

Table 3 and Figure 6 also illustrate and compare the extraction protocols’ performances in quantifying the total yield (in a million bases), read length, read quality, and the number of specific IAV reads generated during the runs.

As all the listed variables are continuous, linear mixed regression models assessed the least squares mean differences between the extraction protocols. The total yield, read length, read quality, and the natural log of the IAV reads were each used as the response or dependent variables in the mixed models, the extraction protocols were the fixed effects, and the tissue type nested within each animal ID was used as the random effect.

## 3. Results

### 3.1. IAV Detection Rate and RNA Concentration

TRIzol demonstrated statistically significant superiority in terms of the mean log concentration of total RNA compared to all other methods across different samples (with a mean log concentration of 202.74 ng/µL) and a lower AIV-specific C_T_ value compared to the magnetic particle-based and enzymatic methods (mean C_T_ of 19.49), as shown in Figure 2 and Figure 3. However, its ability to extract AIV from clinical samples was inconsistent, reflected by its lower detection rate in rRT-PCR results (Figure 4) compared to MagMAX and QIAmp. TRIzol had false negative results in 12 out of 48 extracts, while MagMAX and QIAmp had false negative results in 4 and 3 extracts, respectively. MagMAX and QIAamp performed similarly regarding their total RNA concentration and qPCR results. There was no statistically significant difference between the MagMAX and QIAmp mean log concentration results, with MagMAX showing a mean log concentration of 23.57 ng/µL and QIAmp 21.40 ng/µL. Additionally, there was no statistical difference regarding the MagMAX and QIAmp mean C_T_ values. It was also noteworthy that viral-specific concentration did not follow the same tendency as total concentration (i.e., some samples with a high total RNA concentration showed low AIV copy numbers, as predicted by high C_T_ values rather than low C_T_ values, Supplementary Material, Table S1). Other samples, such as spleen and air sac swabs, had a low total concentration and high AIV copy numbers. This difference may be due to the RNA in clinical samples representing the host rather than viral RNA. SwiftX Swabs consistently performed the worst, showing the lowest mean log concentration and the lowest detection rate by qPCR (with a mean log concentration of 1.8 ng/µL and mean C_T_ of 26.86), statistically inferior to the other protocols in both the evaluated parameters. 

### 3.2. Purity Analysis

Regarding purity evaluation between the protocols, QIAamp exhibited the best performance across different samples, resulting in higher log odds of observing outcomes within the desirable range of 1.9–2.2 of RNA extracts (a probability of 72.66% of the occurrence of a pure sample in our logistic regression model). On the other hand, there was no significant statistical difference in the mean purity ratio between TRIzol and MagMAX (Figure 5), and both presented a similar number of samples classified as “impure” from our analysis. Finally, the Swift Swabs protocol presented the highest number of samples classified as “impure” and statistically lower log odds for the purity analysis (Figure 6).

### 3.3. Sequencing Performance

Although there were no statistical differences among the total yields (i.e., total generated bases from the sequencing run presented in million bases (Mb)) between MagMAX, TRIzol, and Swift Swabs, MagMAX had a higher read count than the other methods, resulting in an average of 46.53 million bases. In contrast, QIAmp showed the lowest yield, averaging 11.07 Mb. 

**Figure 7:**
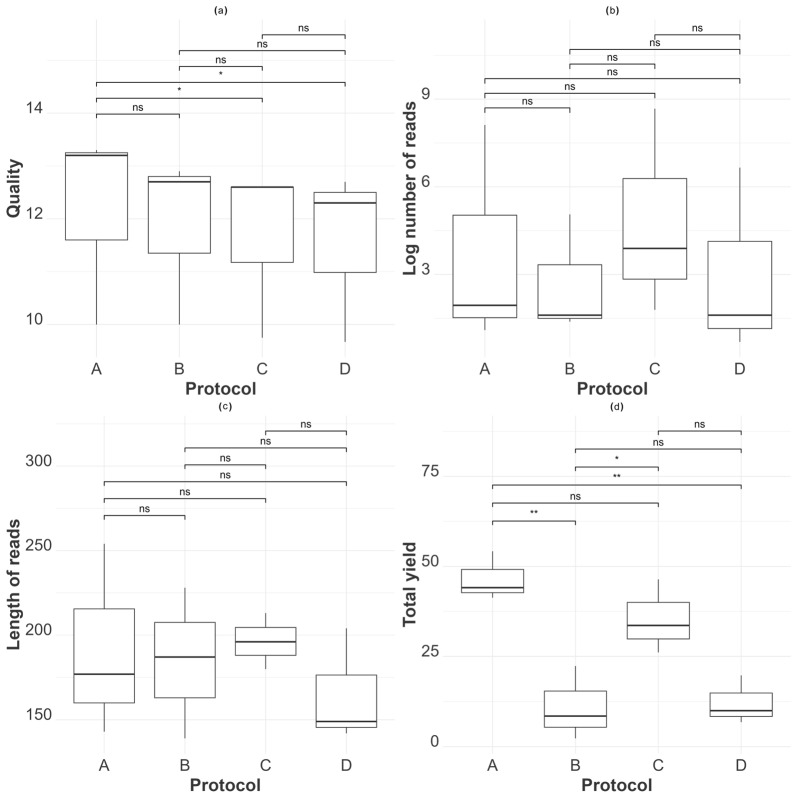
Metagenomic nanopore sequencing results by protocol. A-D plots of the different aspects evaluated to assess the sequence performance of the extraction protocols, represented as MagMAX Pathogen RNA/DNA™ (A), QIAamp^®^ Viral RNA (B), TRIzol™ LS Reagent (C), and SwiftX™ Swabs (D). The boxplots represent the results from the three samples selected for mNGS. (**a**) Quality scores from the generated reads. (**b**) Log-transformed number of Influenza A reads generated. (**c**) Length of the generated reads expressed in bases. (**d**) Total yield (number of total reads) generated by protocol. Statistical differences between protocols are indicated atop the boxplots using the results from a mixed-effect regression model (read quality/log number of reads/read length, yield as the protocol as a fixed effect, and tissue type nested within bird as a random effect). “ns,” “*”, and “**”indicate *p*-values > 0.05, ≤0.05, and ≤0.0001 respectively. An alpha value of 0.05 was used, and *p*-values were adjusted using the Sidak method for six tests.

Although most reads were from the host genome, there was a correlation between the number of total bases and the number of AIV-specific reads generated. As observed for QIAmp (B) and Swift Swabs (D), they showed fewer total bases and fewer logAIV reads (Figure 7d). The summary of the extraction evaluation and sequencing data is expressed in Table 3. 

To assess the fragment length of the sequences, we compared the N50 generated by each protocol using the Nanoplot tool in Galaxy. N50 is a commonly used parameter for assessing the contiguity of ONT-generated sequencing data, and it is defined as the read length that 50% of the reads are equal to or more than. A higher N50 reflects a corresponding increase in the average read length and suggests a greater likelihood of contiguity in the assembly of reads. TRIzol produced longer reads, with a mean N50 of 196 bases, and Swift Swabs, the shortest reads, with a mean N50 of 165 bases. However, the kits had no significant length difference (Figure 7c). 

To address the wide range of AIV reads obtained across the samples, we applied a log transformation to the read counts, compressing higher values and increasing the distribution of small values (Figure 7b). TRIzol showed the highest log-transformed AIV read counts (mean log reads of 4.78 and a median of 3.89). QIAmp produced fewer AIV reads, with the lowest mean AIV log reads of 2.68 and a median of 1.61. 

Finally, to evaluate the accuracy of the generated reads, we assessed the mean read quality score using the WIMP tool. The quality score is based on the Phred score, and across the different samples we evaluated, all four protocols exhibited an average quality score above 12, representing an accuracy higher than 90%. Furthermore, when comparing the mean read quality among the protocols for each sample, there was no statistical difference between their mean quality score, indicating a comparable read quality performance (Figure 7a).

### 3.4. Applicability of Different Extraction Protocols

The extraction procedure applicability was also established as a comparison parameter, considering that we aimed to combine the MinION’s practicality with a suitable extraction workflow for AIV. Applicability in the current study was associated with the simplest, most reproducible method (reducing individual bias while performing the method), cost, flexibility with different tissue types, the equipment needed to execute each protocol, and input/output volumes. 

Overall, MagMAX and Swift Swabs were the least time-consuming, with a reduced hands-on time of 25 min. Despite needing specific extraction equipment (Kingfisher Flex) for MagMAX, it would concomitantly allow the extraction of 96 samples, which is convenient for a diagnostic laboratory setting with a high caseload. The SwiftX Swabs method is advantageous overall because it only requires a regular heat block. In contrast, QIAmp and TRIzol were the most time-consuming; they included sequential centrifuging steps and presented less passive time because they required multiple pipetting and mixing steps. It is noteworthy that QIAmp was performed manually during this study. However, automation of the protocol is possible, which would significantly reduce the time, especially for extracting a high number of samples.

Additionally, there were specific limitations associated with some methods. For instance, the TRIzol method is highly error-prone because it depends on personal decisions during aqueous phase pipetting and pellet visualization, leading to inconsistencies and inaccuracies. Meanhile, Swift Swabs presented a limitation considering the final aspect of the extract; some of the extracts solidified and prevented the separation of a clear solution for further application. This condition might have affected the rRT-PCR results and the purity of the samples extracted by Swift Swabs.

### 3.5. Sample Type Comparison

Considering that different sample types were included in the study, the C_T_ value and rRT-PCR detection rate were evaluated across these samples to assess the extraction protocols’ performance (Table S1 and Figure S2 in the Supplementary Materials). Brain and lung tissues consistently tested positive (detection rate = 1) across different protocols, except for a single brain sample (sample #21) that showed a C_T_ value greater than 40 (detection rate = 0) when extracted with TRIzol (Method C). Notably, brain tissue exhibited the lowest mean C_T_ value across all extraction protocols. TRIzol had a lower detection rate for swab samples (air sac and spleen swabs), with three out of four extracts testing negative in the rRT-PCR test. Additionally, the SwiftX Swabs (Method D) showed a lower detection rate in liver and trachea samples, with all six extracts testing negative in the rRT-PCR test.

## 4. Discussion

As a foreign animal disease (FAD), avian influenza impacts the poultry industry and brings economic and social requirements for its detection and control [1]. The output of a nanopore sequencing run is strongly influenced by all the preceding steps, including the nucleic acid extraction, enrichment, and library preparation steps. Therefore, all the procedures involved in sample preparation for AIV detection and sequencing must be evaluated to establish a reproducible, rapid, and accurate diagnostic workflow using ONT. Extraction is the base step upon which all the other steps for an optimal sequencing run are built, and it is essential for obtaining high-quality nucleic acid. Thus, in this study, we evaluated the extraction step by comparing four commercial extraction kits. The extraction is based on three main steps: the disruption of cells and tissue, denaturation of nucleoprotein complexes, and inactivation of nucleases (DNase and RNase) [39]. After extraction, the target nucleic acid should be in a high concentration and free of contaminants such as proteins, carbohydrates, or lipids [40]. The different principles, reagents, and procedures used by each protocol impact the RNA recovery and purity, interfering with pathogen detection and characterization with NGS. Additionally, input and output sample volume, throughput, automation, and cost differ across the methods, leading to differences in the suitability for diagnostics settings. 

### 4.1. Performance of the Kits—Evaluating the Extracts’ Concentration (Total and Viral-Specific) and Purity

We evaluated the RNA concentration obtained from four extraction methods, categorizing our results into total RNA concentration assessed by fluorometry, and AIV-specific concentration by rRT-PCR. As shown in Figure 3 and Table S1 TRIzol LS (Method C) had the highest RNA recovery efficiency across different tissue types. Other studies comparing RNA concentrations between QIAmp (Method B) and acid guanidium thiocyanate–phenol–chloroform extraction (a similar extraction principle as TRIzol) procedures used different sample types (blood, tissues, exhaled breath, and airborne environment) and revealed similar outcomes [41,42,43]. Although TRIzol had the lowest C_T_ values in tissue samples, it had a lower rRT-PCR detection rate in swab samples than the other kits (Table S1). Knepp et al. (2003) reported comparable results after examining various nucleic acid extraction methods for Enterovirus RNA recovery from cell culture [44]. This difference in the TRIzol LS recovery rate can be attributed to the method’s superior performance with tissue samples but not with relatively cell-free samples such as swabs [45,46]. The inconsistent detection rate of TRIzol LS is a significant limitation of this method in a diagnostic setting, since swabs are the primary sample type for AIV detection and monitoring. 

Our investigation showed that SwiftX™ Swabs’ efficacy in RNA extraction was lower than the other evaluated kits. This led to a reduced recovery rate indicated by a lower log concentration and higher C_T_ values. This protocol has been validated as a tool for RNA extraction from fresh swabs and saliva samples. It is intended to work best when swabs are placed directly into the reagent solution without prior dilution in transport media [47]. In our study, we utilized tissue samples with a high cell concentration, and the swabs were subjected to freezing before extraction. To date, no further studies have assessed the potential of this method for AIV extraction. Hence, additional research is needed before recommending incorporating this approach as a standard diagnostic procedure.

Our study found that the magnetic particle-based method (MagMAX) and the silica column (QIAmp) had similar results in total RNA concentration and rRT-PCR performance for different sample types. However, previous studies have reported that the magnetic bead method was more effective in extracting nucleic acids from fecal samples for gut microbiota profiling [28] and yielded lower C_T_ values for detecting SARS-CoV-2 [48] when compared to the silica method. Nevertheless, in agreement with our PCR detection rates results, the silica column and magnetic particle-based methods presented high agreement between positive and negative results in the previous study, while TRIzol showed an inferior performance in detecting the viral RNA from the cell culture samples tested [48]. Both methods (MagMAX and QIAmp) are widely used in diagnostic settings, and based on our findings, they are effective approaches for RNA recovery from tissue samples.

As measured by the 260/280 ratio, the RNA purity was also evaluated between the different extraction methods. QIAamp (spin column) produced the best results, but that did not directly correlate with better sequence results. Deng et al. (2005) also compared RNA extraction methods regarding purity by assessing A260/A280, and the spin column procedure had the optimal ratio for most of the tested samples when compared to TRIzol LS [41]. However, another study found comparable purity results when assessing the performance of both methods [43]. The differences in purity results between these studies might be related to a higher efficiency achieved during the washing steps for the TRIzol procedure, which increased the purity of the extraction outcome. In addition, avoiding pipetting contaminants accidentally from the interphase would prevent RNA contamination with protein or phenol [49]. Conversely, a decrease in QIAGEN purity might happen due to the silica membrane clogging. This is more frequent when extracting tissue samples with higher amounts of particles/cells, as demonstrated by Muyal et al. 2009, obtaining a lower A260/A280 ratio [43].

### 4.2. Sequencing Performance Evaluation

This study aimed to select the optimum extraction protocol for AIV recovery from clinical samples that would be applied for mNGS with ONT. We compared the sequencing results of three samples extracted using each protocol to complete the evaluation of the four commercial extraction methods. We assessed the total number of reads generated, the number of specific AIV reads, and the sequences’ length and quality to determine the best extraction protocol.

The outcome of ONT sequencing relies on the extracted RNA’s quality and quantity. Our study found that TRIzol had the highest viral concentration, as evidenced by a lower C_T_ value, and resulted in a high number of avian influenza reads. Obtaining a high number of viral reads is crucial, given that the accurate identification and characterization of the pathogen depends on the depth and breadth of coverage, which can be increased with high-throughput sequencing platforms like ONT. Moreover, the higher error rate of ONT compared to Illumina can be overcome by increasing coverage depth, which allows for confident variant calling [32,50]. Therefore, selecting an extraction protocol that yields more AIV reads is necessary. Previous studies have shown the importance of protocol selection to the final AIV genome assembly for NGS outcome, highlighting that the magnetic particle protocol outperformed regarding AIV segments coverage depth [32]. 

Regarding the quality impact on ONT data, we hypothesized that the purity of the original samples would not have significantly affected our results, given that we used a library preparation kit (SQK-PCB109) incorporating a PCR step. PCR can be used to select successfully barcoded nucleic acids and amplify them, which, in turn, can dilute and decrease impurities in the samples. This process may explain why other low-purity samples extracted with MagMax and TRIzol protocols still outperformed QIAgen, which had better purity results. Therefore, the purity of the samples may have been improved during the library preparation step. However, ONT offers a wide range of library preparation kits, and for the PCR-free protocols, the original purity from the samples may play a role in sequencing data generation. 

Although ONT is a long-read NGS platform, the average length of the generated reads was relatively short. This may be due to several factors in our study, including the freezing–thawing process (as the samples were not fresh), the presence of RNases, and multiple pipetting and vortexing steps throughout the process [51,52]. However, given the segmented genome of IAV, fragment length was not a major concern in our study.

The major limitation of our sequencing outcome was the low amount of AIV reads generated from the clinical samples tested. This limitation can be attributed to most nucleic acid in the samples being from the host genome. Furthermore, the lack of target enrichment strategies and host depletion procedures compromised the assay’s sensitivity. Previous research has shown that DNase treatment is necessary for successful AIV RNA sequencing from clinical samples [32]. Additionally, other studies have described various protocols for AIV NGS directly from clinical samples. These protocols include amplicon sequencing [19,23,53] or sequence-independent enrichment [54] approaches to increase the number of viral reads and improve genome coverage. Although mgNGS is known for its poor sensitivity in viral detection, we chose not to apply any enrichment to ensure a fair comparison between methods.

### 4.3. Sample Type Impact

Based on our results, brain samples presented the highest total RNA and specific viral concentration. These findings are explained by the fact that HPAI viral strains have a systemic distribution in chicken visceral organs, and brain neurons are commonly affected [55]. HPAIV had not previously been associated with clinical signs and lesions in wild birds until the H5N1 Eurasian–African lineage altered the pathobiological dynamics of the disease in these species [56]. In our clinical cases, the birds showed neurological signs and microscopic lesions compatible with a systemic viral infection, including brain inflammation. Those findings also support the higher viral load in brain samples. 

Respiratory samples, especially oropharyngeal swabs, are the most common sample type used in the AIV diagnostic routine; therefore, evaluating the extraction step impact in these samples would be more beneficial. Given the limitations of our study, where access to oropharyngeal swabs was unavailable, we conducted the extraction using air sac and spleen swabs instead. These two alternatives share similarities, such as having fewer host cells and serving as tissues suitable for viral detection because HPAIV strains can be systemically distributed throughout the infected birds [2]. In our work, the respiratory tissues showed a high total RNA concentration, except for in the case of Swift Swabs (as shown in Table S1). Furthermore, the swabs had the lowest concentration values, which is explained by less cellularity in this sample type, reducing the amount of host RNA. However, even with a lower total RNA concentration, air sac swabs presented a high viral load, reflected by its low mean C_T_ value obtained with both the MagMAX and QIAmp protocols. TRIzol was unable to detect AIV from air sac swab samples. One possible explanation is that the air sac swab sample type may have a lower cellularity, resulting in less RNA extraction and a smaller pellet forming, which could affect RNA visualization and capture, highlighting a major limitation of TRIzol extraction. Moreover, SwiftX Swabs was less efficient than MagMAX and QIAmp, as evidenced by its higher C_T_ value.

### 4.4. The Applicability of the Kits

Further comparison between the four methods was implemented based on the suitability of the workflow, to assess their applicability as a standard diagnostic procedure. The automated MagMAX presented many advantages, such as removing consecutive centrifugation steps, column separation, and using hazardous chemicals [40]. In addition, it allows the simultaneous extraction of up to 96 samples in a 25 min reaction. Conversely, it had a higher cost per sample and required a specific instrument for its performance. TRIzol and QIAmp, on the other hand, were more time-consuming and did not allow high-throughput viral RNA extraction in our conditions. TRIzol specifically presented critical stages during the manual extraction of the samples, such as the phase separation step and the need for visual pellet formation. In addition, it was laborious and prone to personal bias due to multiple steps, which limits its large-scale routine application [57]. We recognize that QIAmp is also compatible with automated extraction, which would benefit its application in diagnostic laboratories. 

Finally, we identified SwiftX™ Swabs as a promising procedure due to its cost-effectiveness, decreased hands-on time, and minimal steps and equipment required, making it a good candidate for point-of-care diagnostics. However, due to the sample type (tissue samples) we included in the study, the extraction output was unsuitable for AIV detection, considering that after being heated with the extraction solution, some samples clotted (e.g., became solid instead of an aqueous solution), leading to extracts of worse quality and quantity. In addition, the swabs utilized in the study were frozen, and the kit was intended to be performed on fresh swabs, which could explain its inferior performance with the swabs. Notably, there is a different extraction kit offered by Xpedite Diagnostics, SwiftX™ DNA, which could be utilized to extract DNA or RNA from complex (high cellularity) samples. This protocol has additional lysing steps, and a cellular capture based on adding beads suspension and further heat lysis and releasing the nucleic acids in the sample. Additional analysis should be conducted to evaluate the suitability of SwiftX™ DNA or similar technologies for AIV recovery from tissues.

While our study found that TRIzol yielded superior results in total and viral-specific concentrations, it is important to note that this kit had several drawbacks. These disadvantages included inconsistencies in viral detection when using swab samples (commonly used for routine avian influenza virus diagnostics), a lengthy protocol, and susceptibility to personal bias. In contrast, the QIAmp kit excelled in purity but underperformed in various sequencing parameters across different samples. Lastly, although MagMAX did not outperform the other methods in our evaluation, it offers several advantages. It is an automated protocol, allowing for the simultaneous processing of up to 96 samples in the KingFisher. MagMAX demonstrated consistency and reproducibility, with a viral concentration and purity comparable to other methods. Furthermore, MagMAX performed well with the MinION sequencing platform, yielding a high number of total and specific AIV reads.

## 5. Limitations

This study had some limitations. Firstly, the different extraction methods used in this study had varying input and output volumes, which could affect the final RNA concentration. Our primary aim was to evaluate commercially available methods based on the routine protocols established by their manufacturers. Future studies can be performed to compare different input and output volumes to optimize each method further. Secondly, although nasal and cloacal swabs are the most common sample types for AIV diagnosis, they were not the majority of samples tested in this study. This is due to the study’s circumstances (e.g., a veterinary diagnostic laboratory receiving birds for necropsy and submitting swabs for NVSL Laboratory AIV confirmation tests). However, since AIV causes systemic disease in various bird species, testing different tissue types remains valuable for diagnosing this virus and other systemic diseases affecting poultry. Regarding the comparison between the automated MagMAX extraction protocol and other manual procedures, we acknowledge that an automated option is available for the Qiagen protocol, which could enhance both the speed and consistency of the results. However, we included only the automated MagMAX protocol in our study because it was readily available in our laboratory, as well as in other veterinary diagnostic laboratories across the U.S. Lastly, we did not pursue additional host depletion strategies to enhance the sensitivity of the metagenomic NGS (e.g., a higher centrifugation speed and time, DNase and RNase depletion). Consequently, the number of AIV reads from clinical samples using ONT was limited. Therefore, it was not possible to properly compare the final mapping or assembly results obtained from each RNA sample. It is possible that extending the sequencing run could have improved the recovery of the avian influenza virus genome from the samples; however, this was not performed in the current study. Further testing with host depletion approaches is necessary to improve mNGS for more effective virus identification and characterization.

## 6. Conclusions

None of the protocols evaluated was superior for all the aspects assessed in this study. Different choices regarding the suitability of each method in different situations could be made based on the data presented in this manuscript, which highlights the advantages and disadvantages for each method. However, in our hands, the MagMAX™ Pathogen RNA/DNA Kit overall had many advantages in its applicability (speed and scalability) and consistent sequencing performance (yield, compatible number of AIV-specific reads, and segment length) that would allow it to be routinely used for the first step of an NGS workflow. TRIzol™ LS Reagent performed better regarding the RNA and viral concentration, while the best purity ratios were obtained with QIAamp^®^ Viral RNA, but both kits encountered issues regarding their ease of use and speed. Finally, although SwiftX™ Swabs had promising performance features related to its simplified processing steps and time, the performance was not comparable to the other protocols. We recognize the limitation of not using swabs as the primary sample type in our study, given the significance of this sample type for AIV diagnosis, and the fact that SwiftX™ swabs were specifically designed for use with such samples. We emphasize that our findings were related specifically to AIV samples, and these methods could perform differently when used for different pathogens that are not segmented or have longer genome sizes; therefore, they should be evaluated based on the intended target.

## Figures and Tables

**Table 1:** Description of samples used in the study.

Sample ID	Sample Type	Host	Date of Collection
1	Assorted tissues ^a^	Turkey vulture (*Cathartes aura*)	04/28/2022
2	Brain	Red-tailed hawk (*Buteo jamaicensis*)	04/27/2022
3	Respiratory tissues ^b^	Red-tailed hawk (*Buteo jamaicensis*)	04/27/2022
4	Respiratory tissues ^b^	Great horned owl (*Bubo virginianus*)	04/28/2022
5	Brain	Great horned owl (*Bubo virginianus*)	04/28/2022
6	Brain	Turkey vulture (*Cathartes aura*)	04/28/2022
7	Respiratory tissues ^b^	Turkey vulture (*Cathartes aura*)	04/28/2022
8	Brain	Great horned owl (*Bubo virginianus*)	05/12/2022
9	Respiratory tissues ^b^	Great horned owl (*Bubo virginianus*)	05/12/2022
10	Intestine	American green-winged teal (*Anas crecca carolinensis*)	04/26/2022
11	Respiratory tissues ^b^	American green-winged teal (*Anas crecca carolinensis*)	04/26/2022
12	Intestine	Falcon	04/18/2022
13	Respiratory tissues ^b^	Falcon	04/18/2022
14	Trachea	Chicken (*Gallus gallus domesticus*)	04/01/2022
15	Lung	Chicken (*Gallus gallus domesticus*)	04/01/2022
16	Brain	Chicken (*Gallus gallus domesticus*)	04/01/2022
17	Spleen swabs	Chicken (*Gallus gallus domesticus*)	04/01/2022
18	Air sac swabs	Chicken (*Gallus gallus domesticus*)	04/01/2022
19	Liver	Eagle (*Haliaeetus leucocephalus*)	05/01/2022
20	Trachea	Eagle (*Haliaeetus leucocephalus*)	05/01/2022
21	Brain	Eagle (*Haliaeetus leucocephalus*)	05/01/2022
22	Liver	Eagle (*Haliaeetus leucocephalus*)	05/01/2022
23	Intestine	Eagle (*Haliaeetus leucocephalus*)	05/01/2022
24	Lung	Eagle (*Haliaeetus leucocephalus*)	05/01/2022

^a^ Assorted tissues = combination of multiple organs, including respiratory (lung, trachea), enteric (intestine), and central nervous system. ^b^ Respiratory tissues = combination of trachea and lung.

**Table 2:** Description of the four protocols regarding technical (method, cost, equipment required, automation) and functional aspects (starting and elution volume).

Protocol	Extraction Method	Cost Per Sample (US$)	Equipment Requirement	Automation Potential	Starting Volume (μL)	Elution Volume (μL)
MagMAX Pathogen RNA/DNA™	Magnetic particle-based	4.60	Magnetic robot	Yes	100	90
QIAamp^®^ Viral RNA	Silica membrane	5.50	Centrifuge	Available	140	40–60
TRIzol™ LS Reagent (DNA/RNA)	Liquid-phase separation	1.90	Centrifuge	No	250	50
SwiftX™ Swabs (DNA/RNA)	Enzymatic	1.30	Thermal block	No	100	200

**Table 3:** Description of the selected samples for nanopore sequencing.

Sequencing Sample		1	2	3
Animal ID		CN	GHO	CN
Tissue type		Trachea	Brain	Brain
Protocol A (MagMAX™)	Av conc	9.8	32.5	35.7
	C_T_	18.8	11.4	23.0
	Purity	1.6	2.1	2.0
	Total number of reads (Mb)	41.3	44.1	54.2
	AIV reads (count)	7	3340	3
	N50 (bases)	177	143	254
Protocol B (QIAamp^®^ Viral RNA)	Av conc	21.8	15.55	66.5
	C_T_	19.3	12.9	19.5
	Purity	1.9	2.0	1.9
	Total number of reads (Mb)	8.5	2.3	22.4
	AIV reads (count)	5	157	4
	N50 (bases)	187	139	228
Protocol C (TRIzol™ LS Reagent)	Av conc	368	1975	985
	C_T_	17.4	6.9	18.8
	Purity	1.8	2.0	1.9
	Total number of reads (Mb)	26.1	46.4	33.6
	AIV reads (count)	49	5833	6
	N50	213	180	196
Protocol D (SwiftX™ Swabs)	Av conc	9.5	41.5	20.6
	C_T_	27.2	13.2	25.3
	Purity	2.1	1.5	1.6
	Total number of reads (Mb)	6.8	19.8	10
	AIV reads (count)	5	778	2
	N50	149	142	204

CN: chicken. GHO: great horned owl.

## Data Availability

The raw data presented in this study are openly available in the SRA under the accession numbers SRR27468981, SRR27468980, SRR27468979, SRR27468978, SRR27468977, SRR27468976, SRR27468975, SRR27468974, SRR27468973, SRR27468972, SRR27468971, SRR27468970, and the BioProject accession number PRJNA1062777.

## Supplementary Materials

The following supporting information can be downloaded at: https://www.mdpi.com/article/10.3390/v16091429/s1, Table S1: Mean total RNA concentration, expressed in nanograms per microliters, and C_T_ value from each sample by protocol. Samples are ordered according to the animal species, and number IDs are added corresponding to the numbering described in Table 1; Figure S1: Mean cycle threshold (C_T_ values, log concentration, and purity for each protocol in the two experimental sessions (1 and 2); Figure S2: The count of positive samples by rRT-PCR by sample type and by extraction protocol, represented as MagMAX Pathogen RNA/DNA™ (A), QIAamp^®^ Viral RNA (B), TRIzol™ LS Reagent (C), and SwiftX™ Swabs (D). An extract has a positive detection (1) when the C_T_ value is less than (<) 40 and a negative detection (0) when the C_T_ value is equal to or higher than 40. AGT: American green-winged teal. CN: chicken. EE: eagle. FN: falcon. GHO: great horned owl. HK: hawk. TV: turkey vulture.
