# Supplementary material for: Evaluation of Commercial RNA Extraction Protocols for Avian Influenza Virus Using Nanopore Metagenomic Sequencing
Source: Viruses. 2024 Sep 7;16(9):1429. doi: 10.3390/v16091429 (PMC11437427; doi:10.3390/v16091429)
Supplement: Supplementary file 1 [file viruses-16-01429-s001.zip › viruses-3148644-supplementary.pdf]

viruses

MDPI

Supplementary materials

Table S1: Mean total RNA concentration, expressed in nanograms per microliters, and C_T value from each sample by protocol represented as MagMAX Pathogen RNA/DNA ™ (A), QIAamp® Viral RNA (B), TRIzol™ LS Reagent (C), and SwiftX™ Swabs (D). Samples were ordered according to the animal species, and corresponding numbers ID were added corresponding to the numbering described in Table 1.

**Protocol**			**A**		**B**		**C**		**D**	
**Animal ID**	**Sample type**	**Sample ID**	**Mean conc**	**C_T value**	**Mean conc**	**C_T value**	**Mean conc**	**C_T value**	**Mean conc**	**C_T value**
AGT	Intestine	10	15.18	> 40	109.74	31.60	23.93	32.19	5.57	> 40
	Respiratory	11	24.43	37.60	23.53	39.36	321.50	29.79	4.40	> 40
CN	Air sac swabs	18	0.25	20.72	0.25	19.93	0.25	> 40	0.25	28.07
	Brain	16	40.00	20.23	70.25	17.91	1087.5	16.97	26.68	23.42
	Lung	15	8.12	19.11	9.41	19.26	275.00	13.74	0.25	26.29
	spleen swabs	17	1.88	22.24	4.52	21.20	6.02	21.90	0.25	31.72
	Trachea	14	9.52	20.81	18.03	20.90	233.50	18.80	7.36	28.19
EE	Brain	21	14.11	19.59	14.35	19.28	95.25	19.52	0.25	29.93
	Intestine	23	18.16	27.89	29.93	27.52	105.18	33.08	14.03	25.97
	Liver	19	405.75	28.92	275.25	28.58	2182.5	26.78	2.62	> 40
	Liver	22	200.18	24.62	64.03	23.62	865.75	22.32	0.25	> 40
	Lung	24	39.73	25.83	71.18	24.55	715.00	23.93	0.25	38.13
	Trachea	20	30.35	27.64	29.45	27.41	224.50	26.39	2.47	> 40
FN	Intestine	12	9.62	26.64	12.76	26.39	75.25	25.13	3.79	34.07
	Respiratory	13	13.00	26.78	16.85	25.82	189.25	25.59	3.86	35.35
GHO	Brain	5	28.70	10.43	13.10	13.05	1942.5	7.15	31.55	13.24
	Respiratory	4	54.25	16.03	60.50	15.05	467.50	14.70	1.18	28.36
GHO2	Brain	8	53.25	11.23	23.40	13.97	1957.5	7.49	48.20	12.23
	Respiratory	9	66.43	19.16	128.75	18.04	1150.0	17.62	0.25	27.56
HK	Brain	2	21.60	11.47	15.21	15.60	975.00	7.51	24.45	14.61
	Respiratory	3	41.20	13.35	48.83	12.54	321.50	12.64	0.25	30.99
TV	Brain	6	16.77	22.21	47.75	21.47	350.00	19.97	4.81	28.11
	Assorted	1	14.55	> 40	25.30	38.54	876.50	> 40	0.25	31.60
	Respiratory	7	14.48	19.18	20.53	18.20	100.00	18.71	1.18	28.66

AGT: American green-winged teal. CN: Chicken. EE: Eagle. FN: Falcon. GHO: Great horned owl. HK: Hawk. TV: Turkey vulture

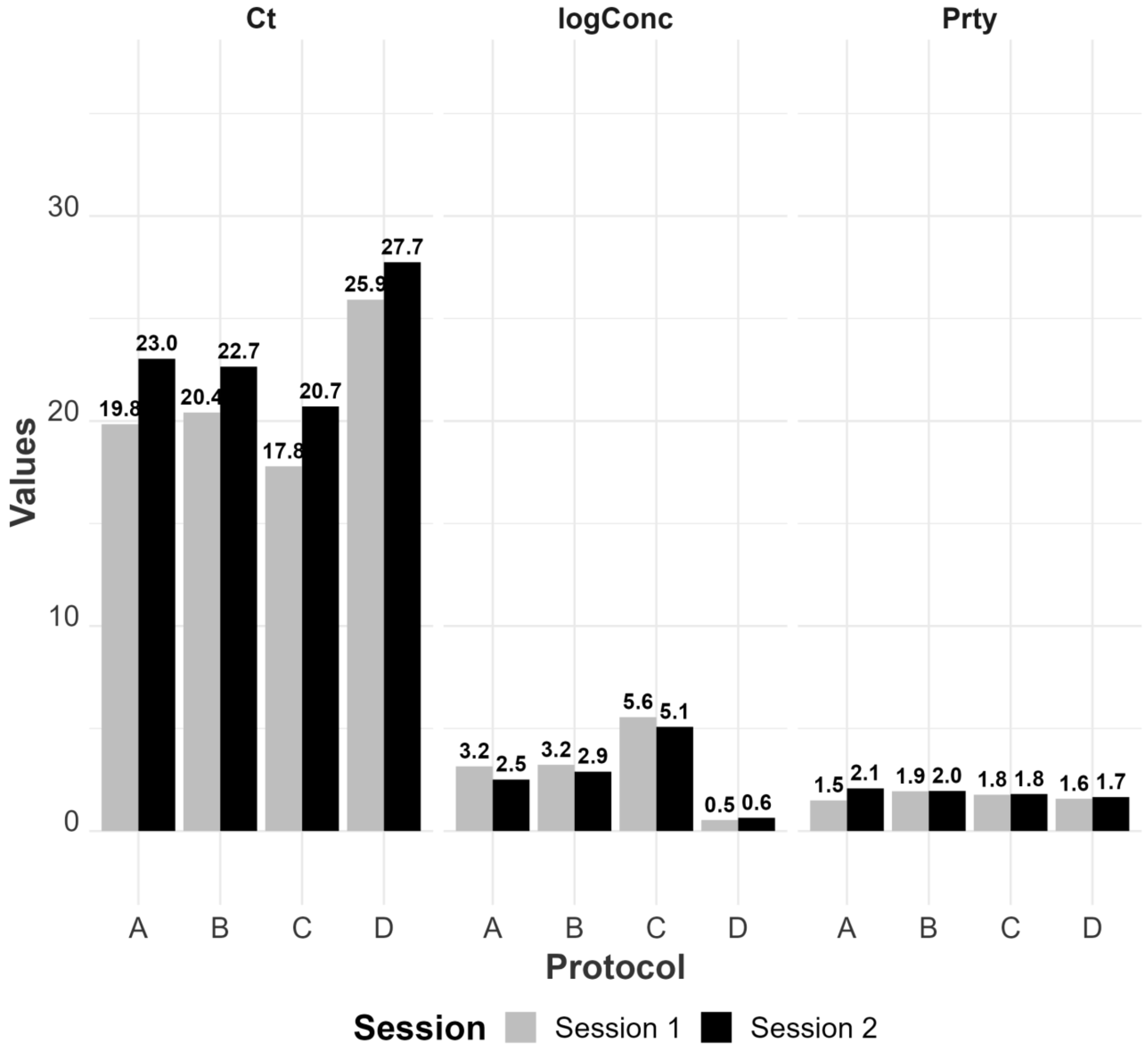

Figure S1: Mean values of Cycle threshold (C_T) values, Log concentration, and purity for each protocol in each of the two experimental sessions (1 and 2).

viruses

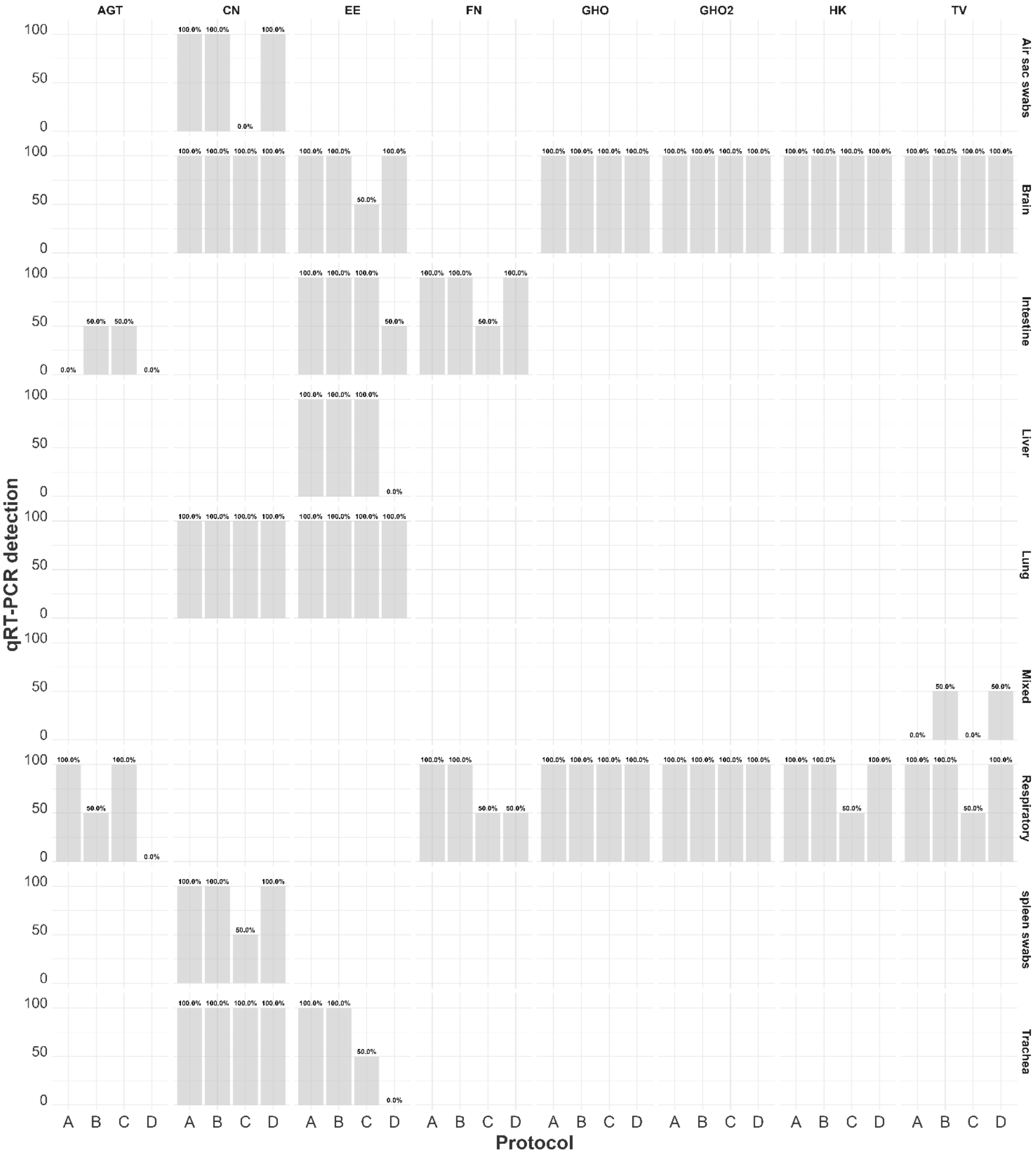

Figure S2: The count of positive samples by qRT-PCR by sample type and by extraction protocol, represented as MagMAX Pathogen RNA/DNA ™ (A), QIAamp® Viral RNA (B), TRIzol™ LS Reagent (C), and SwiftX™ Swabs (D). An extract has a positive detection (1) when the C_T value is less than 40 and a negative detection (0) when the C_T value is equal to or higher than 40. The bars represent the mean detection percentage per protocol according to the sample type and bird species. AGT: American green-winged teal. CN: Chicken. EE: Eagle. FN: Falcon. GHO: Great horned owl. HK: Hawk. TV: Turkey vulture
